# Foetal weight prediction models at a given gestational age in the absence of ultrasound facilities: application in Indonesia

**DOI:** 10.1186/s12884-018-2047-z

**Published:** 2018-11-06

**Authors:** Dewi Anggraini, Mali Abdollahian, Kaye Marion

**Affiliations:** 1 School of Science (Mathematical and Geospatial Sciences), College of Science, Engineering, and Health, RMIT University, GPO BOX 2476, Melbourne, VIC 3001 Australia; 2 Study Program of Statistics, Faculty of Mathematics and Natural Sciences, University of Lambung Mangkurat (ULM), Ahmad Yani Street, Km. 36, Banjarbaru, South Kalimantan 70714 Indonesia

**Keywords:** Fundal height, Gestational age, Estimated foetal head circumference, Estimated foetal abdominal circumference, Regression analysis, Foetal weight estimation, Absence of ultrasound facilities, Primary health care centre, Prediction accuracy, Indonesia

## Abstract

**Background:**

Birth weight is one of the most important indicators of neonatal survival. A reliable estimate of foetal weight at different stages of pregnancy would facilitate intervention plans for medical practitioners to prevent the risk of low birth weight delivery. This study has developed reliable models to more accurately predict estimated foetal weight at a given gestation age in the absence of ultrasound facilities.

**Methods:**

A primary health care centre was involved in collecting retrospective non-identified Indonesian data. The best subset model selection criteria, coefficient of determination, standard deviation, variance inflation factor, Mallows C_p_, and diagnostic tests of residuals were deployed to select the most significant independent variables. Simple and multivariate linear regressions were used to develop the proposed models. The efficacy of models for predicting foetal weight at a given gestational age was assessed using multi-prediction accuracy measures.

**Results:**

Four weight prediction models based on fundal height and its combinations with gestational age (between 32 and 41 weeks) and ultrasonic estimates of foetal head circumference and foetal abdominal circumference have been developed. Multiple comparison criteria show that the proposed models were more accurate than the existing models (mean prediction errors between − 0.2 and 2.4 g and median absolute percentage errors between 4.1 and 4.2%) in predicting foetal weight at a given gestational age (between 35 and 41 weeks).

**Conclusions:**

This research has developed models to more accurately predict estimated foetal weight at a given gestational age in the absence of ultrasound machines and trained ultra-sonographers. The efficacy of the models was assessed using retrospective data. The results show that the proposed models produced less error than the existing clinical and ultrasonic models. This research has resulted in the development of models where ultrasound facilities do not exist, to predict the estimated foetal weight at varying gestational age. This would promote the development of foetal inter growth charts, which are currently unavailable in Indonesian primary health care systems. Consistent monitoring of foetal growth would alleviate the risk of having inter growth abnormalities, such as low birth weight that is the most leading factor of neonatal mortality.

**Electronic supplementary material:**

The online version of this article (10.1186/s12884-018-2047-z) contains supplementary material, which is available to authorized users.

## Background

Birth weight is a primary measurement and significant indicator to ensure the optimal growth, survival, and future well-being of new-borns. Deviation from normal delivery weights (2500–3999 g), such as low birth weight (LBW) (< 2500 g) and macrosomia (> 4000 g) could lead to some negative consequences on neonatal health [1–3]. While macrosomia may cause neonatal and maternal morbidity [4], LBW is well-documented to be one of the most contributing factors to the neonatal mortality [1]. LBW is defined as weight less than 2500 g at birth regardless of gestational age (GA) and can be caused by preterm birth or intrauterine growth restriction [5]. In this paper, LBW includes both preterm and term new-borns of appropriate for GA.

Routine and reliable estimates of foetal weight at a given GA throughout pregnancy are vital. These estimates could create evidence-based track records/analysis to assist medical practitioners to detect the signs of potential LBW during pregnancy and provide the appropriate interventions. Although a wide range of simple and advanced multivariate weight prediction models based on clinical and ultrasonic measurements has been developed, most are only based on maternal or foetal factors [6–25]. Less is known about the combinations of these characteristics to estimate foetal weight during pregnancy despite the fact that birth weight is significantly associated with characteristics of both mother and foetus [1, 26].

Several models based on combined maternal and neonatal characteristics have been developed and reviewed, these existing models were mostly developed based on the information available at delivery time [27, 28]. In most developing countries, the availability of foetal biometric measurements during pregnancy is low, particularly in rural areas due to limited access to ultrasound machines and skilled personnel [29]. Westerway et al. (2000), Loughna et al. (2009), and Papageorghiou et al. (2014) have used a large number of ultrasonic measurements to develop formulas that estimate foetal biometric characteristics at a given GA [30–32]. These formulas then could be used to fill the foetal database gaps during pregnancy when ultrasound facility is absent.

The present research develops foetal weight prediction models based on combined maternal and estimated foetal biometric characteristics to estimate foetal weight at any given GA. The proposed models can be simply implemented in low-resource primary health care centres where ultrasound machines and trained ultra-sonographers are not always available. The predicted foetal weight will assist in the development of foetal growth charts for Indonesia. No such charts currently exist for the Indonesian population.

## Methods

### Study design and setting

A quantitative and analytic study based on a retrospective pregnancy cohort analysis was carried out. Unidentified secondary quantitative data were collected and analysed to (1) assess the adequacy of the existing ultrasonic models in estimating foetal biometric characteristics, (2) develop new foetal weight prediction models based on both maternal and estimated foetal biometric characteristics, (3) assess the accuracy of the proposed models in predicting foetal weight between 35 and 41 weeks of GA, and (4) carry out a comparison study between the proposed and commonly used models. The study was conducted in a primary health care centre in South Kalimantan province, Indonesia. The locality was selected because it is one of the five provinces with the highest neonatal mortality in the country [33–35].

### Conceptual framework

**Fig. 1:**
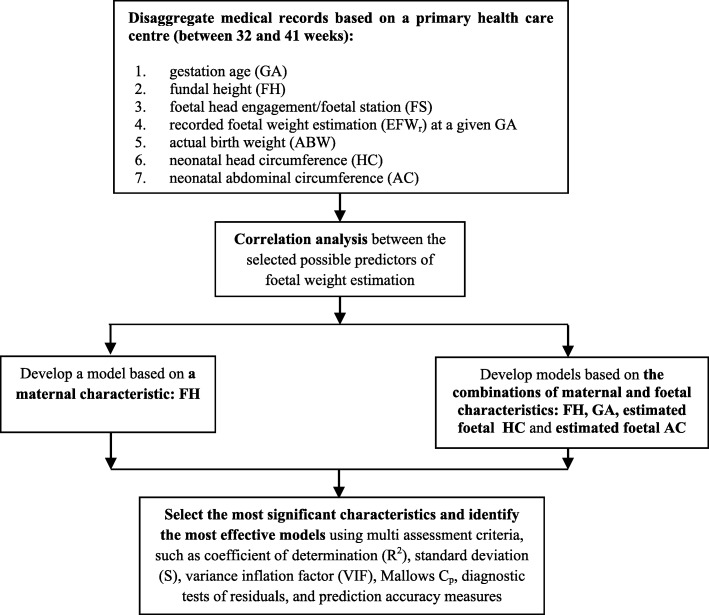
Conceptual framework for factors influencing foetal weight estimation between 32 and 41 weeks of pregnancy

Figure 1 shows the framework used in this study, along with the selected possible predictors of foetal weight estimation.

### Data source

Study data were sourced primarily from a paper-based pregnancy register of pregnant women who received antenatal care (ANC) services and gave birth in the selected primary health care centre from January 2013 to August 2015. Prior to delivery, GA, fundal height (FH), foetal head engagement/foetal station (FS), and recorded foetal weight estimation (EFW_r_) at a given GA were measured and recorded by the assigned midwives. At delivery time, actual birth weight (ABW), neonatal head circumference (HC), and neonatal abdominal circumference (AC) were also measured and recorded.

### Data management

Data was recorded in Microsoft Excel and the statistical analyses were performed using Minitab version 17 and R. The ordinary least square (OLS) and robust regression (the weighted likelihood estimation) were carried out by using ***lm*** function and ***wle.lm*** function, respectively in R [36–39].

### Statistical analysis

#### The adequacy assessment of existing ultrasonic models to estimate foetal biometric characteristics during pregnancy

The existing ultrasonic formulas to estimate foetal HC and foetal AC which were developed based on the Australian foetal biometry data (measured between 11 and 41 weeks), the UK foetal biometry data (measured between 13 and 42 weeks), and the international foetal biometry data (measured between 14 and 42 weeks or until birth) [30–32] (provided in Additional file 1: Table S1) were applied to estimate foetal HC and foetal AC at a given GA for Indonesian foetus (*n* = 127). A reliability analysis using intraclass correlation coefficient (ICC) [40, 41] was performed to assess the consistency of the ultrasonic formulas for Indonesian population. The obtained ICC values (provided in Additional file 2: Table S2) were computed by single-rating, consistency, and two-way random effects models for the foetal biometrics with three raters (different ultrasonic formulas) across 127 subjects (pregnant women). Interclass (Pearson) correlation coefficient was also analysed to assess whether there is a significant relationship between the predicted foetal biometrics and the neonatal measurements recorded at delivery time (provided in Additional file 3: Table S3).

#### The development of new foetal weight prediction models based on combinations of maternal and estimated foetal biometric characteristics

Bernoulli distribution with the event probability (p) of 70% was used to randomly divide our data into two sets: model development (training) data (*n* = 89) and model efficacy assessment (testing) data (*n* = 38).

Based on the training data set, simple and multivariate linear regressions were used to develop our proposed models. The best subset selection methodology together with correlation coefficient (*r*), coefficient of determination (*R*^2^), standard deviation (*S*), Mallows C_p_, and variance inflation factor (*VIF*) were deployed to identify the most suitable subset of predictors. Analysis of variance (ANOVA) together with t-test statistics was used to simultaneously and partially confirm the significance of predictors’ contribution in the regression models. Diagnostic tests of residuals were used to confirm the validity of the regression models.

Since our aim is to investigate whether a combination of maternal and foetal factors could improve foetal weight prediction accuracy, we have utilised the most commonly recommended formulas of ultrasonic foetal measurement standards (based on GA) to predict the measurements of foetal biometrics in our local population. This prediction is one way to fill in the foetal database gaps during pregnancy in the absence of ultrasound. The estimates of these two most significant characteristics of foetal biometry, such as HC and AC were then combined with maternal FH to develop the prediction models. The idea of this combination was to evaluate whether it could improve the prediction accuracy of foetal weight.

Our delivery date in our data ranged from 32 to 41 weeks. The ultrasonic formulas were deployed to estimate foetal HC and foetal AC at the given GA for each individual patient and used to estimate the delivery weight. Therefore, the mean time between the last measurements of FH and GA as well as the last estimates of foetal HC and foetal AC and birth was assumed to be 0 days.

#### The efficacy assessment of the proposed models

The testing data set was used to validate and assess the efficacy of the proposed models. The potential bias due to growth between the last measurements and birth of the developed models for estimating foetal weight was assessed by calculating the mean prediction error [the average of the differences between the *i*^*th*^ actual values of birth weight (*ABW*_*i*_) and the *i*^*th*^ predicted values of foetal weight based on the proposed models (${EFW}_{p_i}$)]=$\sum \limits_{i=1}^n\frac{\left({ABW}_i-{EFW}_{p_i}\right)}{n}$. The mean absolute percentage prediction error or $MAPE=\sum \limits_{i=1}^n\frac{\left|\left(\frac{ABW_i-{EFW}_{p_i}}{ABW_i}\times 100\right)\right|}{n}$ was also calculated to represent the dispersion of the errors [42]. In addition, the median absolute percentage prediction error or $MEDAPE= Median\ \left|\left(\frac{ABW_i-{EFW}_{p_i}}{ABW_i}\times 100\right)\right|$ was measured and used for assessing the efficacy of the models. The later measurement is more resistant to outlier distortion (due to the presence of extreme deviations) than the mean; therefore, deploying *MEDAPE* would eliminate the false interpretation of forecast accuracy [43].

The efficacy of the proposed models was also assessed by the number of estimates within 10% of ABW. A two independent sample t-test was used to decide if there is a significant difference between the observed or actual values of birth weight (ABW), EFW_r_, and estimated foetal weights based on the proposed models (EFW_p_). Multiple comparisons were carried out between our proposed models, eleven existing clinical models, and six existing ultrasonic models to select the most effective models for estimating foetal weights at a given GA.

## Results

**Fig. 2:**
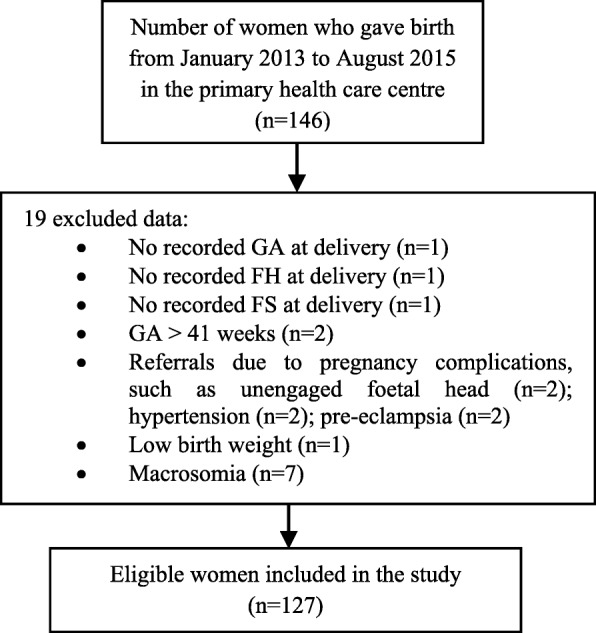
Flowchart of recruitment of participants through the study

Out of 146 women who received ANC services and gave birth in the selected primary health care centre, 127 (87%) women met the study criteria (Fig. 2). These women delivered live singletons with normal delivery weights between 32 and 41 weeks of GA. We excluded 19 (13%) women due to incomplete information on the required characteristics listed in Fig. 2, such as no records of GA, FH, and FS (*n* = 3 ), GA > 41 weeks (*n* = 2), been referred to hospitals due to pregnancy complications (*n* = 6), and abnormal birth weight babies (*n* = 8).

### General information on the study population

Descriptive statistics on baseline characteristics of mother and new-born of the study population (*n* = 127) are presented in Table 1. Overall, the pregnant women were well-nourished (arm circumference = 25.5 cm) and had normal haemoglobin level (11.6 g/dl) and body mass index (24.4 kg/m^2^). The median age, height, weight, and FH for women were 28 years (range 16–44 years), 156 cm (range 148–176 cm), 60 kg (range 44–83 kg), and 32 cm (range 27–36 cm), respectively. The outcomes of pregnancy were in a normal average of GA (38 weeks), delivery weight (3252.8 g), birth length (50.2 cm), neonatal HC (33.5 cm), and neonatal AC (34.5 cm).

**Table 1:** Maternal and neonatal baseline characteristics of study population (*n* = 127)

Characteristics	Missing data	Mean	Standard deviation	Median	Minimum	Maximum
Maternal age (years)	–	27.6	4.9	28	16	44
Maternal height (cm)	3	156.5	5.0	156	148	176
Maternal weight (kg)	–	59.9	7.5	60	44	83
Maternal body mass index (kg/m^2^)	3	24.4	3.1	24.3	16.5	34.2
Maternal arm circumference (cm)	1	25.5	1.7	25	22	31
Maternal haemoglobin level (g/dl)	–	11.6	0.7	11.4	9	13.2
Maternal fundal height (FH) at delivery time (cm)	–	32.2	2.4	32	27	36
Gestational age (GA) at delivery time (weeks)	–	38.6	1.5	39	32	41
Actual birth weight (ABW) (g)	–	3252.8	340.8	3300	2600	4000
Neonatal birth length (cm)	–	50.2	2	50	40	56
Neonatal head circumference (HC) (cm)	–	33.5	1.3	33	29	37
Neonatal abdominal circumference (AC) (cm)	–	34.5	1.9	35	28	37

### The reliability assessment of existing ultrasonic formulas in estimating foetal biometrics

This section presents the results of reliability analysis among three existing ultrasonic formulas [30–32] listed in Additional file 1: Table S1 in predicting foetal biometrics when ultrasound facilities are not accessible.

The intraclass and interclass correlation coefficient analyses are presented in Additional files 2 and 3: Table S2 and S3, respectively. The results presented in Additional file 2: Table S2 indicate that all three formulas have excellent reliability/consistency in predicting foetal HC and foetal AC at a given GA (the obtained ICC values are 0.957 and 0.996, respectively). Therefore, either of the existing formulas can be deployed in our study population.

Additional file 3: Table S3 shows that the estimated ultrasonic HC has a significant relationship with the neonatal HC (*p*-value < 0.0005) based on the existing models although the relationship was weak (0.191 < *r* < 0.212). Meanwhile, there is no significant correlation between the estimated ultrasonic AC and the neonatal AC (0.076 < *r* < 0.078, *p*-value > 0.05). However, since the Australian standard formulas produced slightly higher interclass correlation coefficients (between the estimates of foetal biometrics and the neonatal measurements) and more estimates falling within 10% of the neonatal measurements; therefore, the ultrasonic formulas based on the Australian population will be deployed to fill the foetal database gaps and assist the development of our proposed models.

### Correlation analysis

Prior to developing models, correlations between the potential predictors of foetal weight estimation based on 127 data were investigated. The correlation analysis is presented in Additional file 4: Table S4.

Additional file 4: Table S4 shows that maternal FH has a significant correlation with the EFW_r_ and the ABW (*r* = 0.952, *p*-value < 0.0005 and *r* = 0.795, *p*-value < 0.0005, respectively). Unlike FH, GA has no significant correlation with the EFW_r_ and ABW.

### Optimal models based on the best subset selection algorithm

Deploying the best subset selection algorithm, we have summarised the models developed based on the EFW_r_ (provided in Additional file 5: Table S5). These models were based on one, two, and three independent variables. The table also lists their corresponding *R*^2^, Mallows *Cp*, *S*, and *VIF* statistics.

Additional file 5: Table S5 shows that the first model incorporated only one predictor: FH. The second, third, and fourth models incorporated two predictors: FH and GA, FH and estimated foetal HC, and FH and estimated foetal AC, respectively. The last model was developed based on three predictors: FH, estimated foetal HC, and estimated foetal AC.

Overall, the developed models had equal capability in predicting foetal weight estimation (coefficient of determination between 88.3 and 88.8%). However, using Mallows *Cp* index and *S*, we concluded that Models (3) and (4) were the best fit models with the least predicting errors. Models based only on estimated foetal HC or estimated foetal AC was excluded from the analysis due to the insignificant *R*^2^. Model (5) was excluded due to the presence of severe multicollinearity (*VIF* > 193) (provided in Additional file 5: Table S5).

Table 2 presents the coefficients of the predictors for the chosen models together with the corresponding *p*-values of ANOVA, t-test statistics, and diagnostics of residuals.

**Table 2:** Predictor analysis of the proposed models

Model	Parameters	Estimated coefficients	Simultaneous *p*-value (ANOVA)	Partial *p*-value (t-test)	VIF	Residuals
(1)	β_0_ (Intercept)	− 1538.3	< 0.0005^***^	2.66e-12^***^	–	Non-normal (*p*-value < 0.005)
	β_1_ (FH)	150. 3		< 2e-16^***^	–	
(2)	β_0_ (Intercept)	− 959	< 0.0005^***^	0.011^*^		Non-normal (*p*-value < 0.005)
	β_1_ (GA)	−15.8		0.071^*^	1.01	
	β_2_ (FH)	151.2		< 0.0005^***^	1.01	
(3)	β_0_ (Intercept)	− 634.3	< 0.0005^***^	0.2304	–	Non-normal (*p*-value < 0.005)
	β_1_ (FH)	151.2		< 2e-16^***^	1.01	
	β_2_ (estimated HC)	−2.8		0.0682^*^	1.01	
(4)	β_0_ (Intercept)	−996.8	< 0.0005^***^	0.00548^**^	–	Non-normal (*p*-value < 0.005)
	β_1_ (FH)	151.2		< 2e-16^***^	1.01	
	β_2_ (estimated AC)	−1.6		0.07066^*^	1.01	

^***^Significant at *p*-value < 0.0005

^**^Significant at *p*-value < 0.05

^*^Significant at alpha *p*-value < 0.1

Table 2 shows that for each individual model, the *p*-value corresponding to independent predictors is significant. Since our sample size is large, statistically significant non-normality of residuals was accepted. However, the authors have deployed robust regression to find the best fit models. Unfortunately, the best fit models proposed by robust regression had slightly larger prediction errors than those selected through the best subset models. Therefore, our further analysis is carried out using the OLS regression models presented in Table 2.

### The accuracy comparison of the proposed and existing models

The two most commonly used models in Indonesia for estimating delivery weight are the Johnson-Toshach and the Risanto models. Both models estimate foetal weight based on FH. However, the Johnson-Toshach formula, which is nationally well-recognised, requires additional information on the status of the FS [44].

As listed in Table 2, the first model recommended through the best selection algorithm was Model (1) which is also developed based on FH only. Therefore, the authors carried out further comparisons between the proposed Model (1) and the widely used the Johnson-Toshach [14, 15] and the Risanto models [22, 23] as well as other existing models for estimating foetal weight based only on FH (the Niswander, the modified Niswander, the Mhaskar, the Gayatri-Afiyanti, the Buchmann-Tlale, the Santjaka-Handayani, the Mongelli-Gardosi, and the Yiheyis [16–18, 22–25, 45]). We also compared Models (2), (3), and (4) with the existing models based on ultrasonic measurements of foetal biometrics, such as foetal HC and foetal AC (the Jordaan, the Weiner, the Hadlock 1984, and the Stirnemann [10, 42, 46, 47]). Details for the proposed and existing models are presented in Additional file 6: Table S6.

The prediction accuracy of the proposed (Models (1), (2), (3), and (4) in Table 2) and the existing models were assessed using the testing data set. The predicting errors were calculated as the mean prediction error (the average of the differences between *ABW*_*i*_ and ${EFW}_{p_i}$), the *MAPE*, and the *MEDAPE*. The results are presented in Table 3.

**Table 3:** Accuracy comparisons between the proposed and existing models

Sample size *n* = 38					
(ABW - EFW_p_)	Mean prediction error (g)	MAPE (%)	MEDAPE (%)	Error distribution	Number of estimates within 10% of ABW (%)
Our recommended models					
Model (1)	2.42	5.01	4.10	Normal (*p*-value > 0.05)	92
Model (2)	−0.20	5.10	4.16		92
Model (3)	−1.62	5.10	4.22		89
Model (4)	−0.29	5.10	4.16		92
Existing clinical models					
Johnson (1957) [15]	31.18	5.28	4.73	Normal (*p*-value > 0.05)	89
Risanto I (1995) [22]	149.56	5.95	5.37		84
Risanto II (2014) [23]	152.37	6.00	5.45		84
Niswander (1970) [16]	400.95	12.24	12.07		37
Mod Niswander (1999) [17]	457.68	13.70	14.16		29
Mhaskar (2003) cited in [65]	405.26	12.59	12.86		32
Gayatri (2006) [24]	471.05	14.02	15.15		26
Buchmann-Tlale (2009) [18, 66]	571.05	17.12	18.18		11
Santjaka (2011) [25]	− 2411.95	75.33	72.24		0
Mongelli-Gardosi (2004) [19]	1348.35	41.93	42.40		0
Yiheyis (2016) [45]	363.95	11.12	11.05		45
Existing ultrasonic models					
Jordaan (1983) [46]	− 277.09	14.64	14.43	Normal (*p*-value > 0.05)	39
Weiner II (1985) cited in [60]	486.29	15.90	12.86		32
Hadlock 1984 [10]	−96.83	12.67	12.64		45
Hadlock 1991 [67]	− 42.75	11.74	9.88		50
Stirnemann 2016 [42]	−31.46	12.20	10.88		39
Sotiriadis 2017 [68]	230.72	10.88	9.43		50

**Fig. 3:**
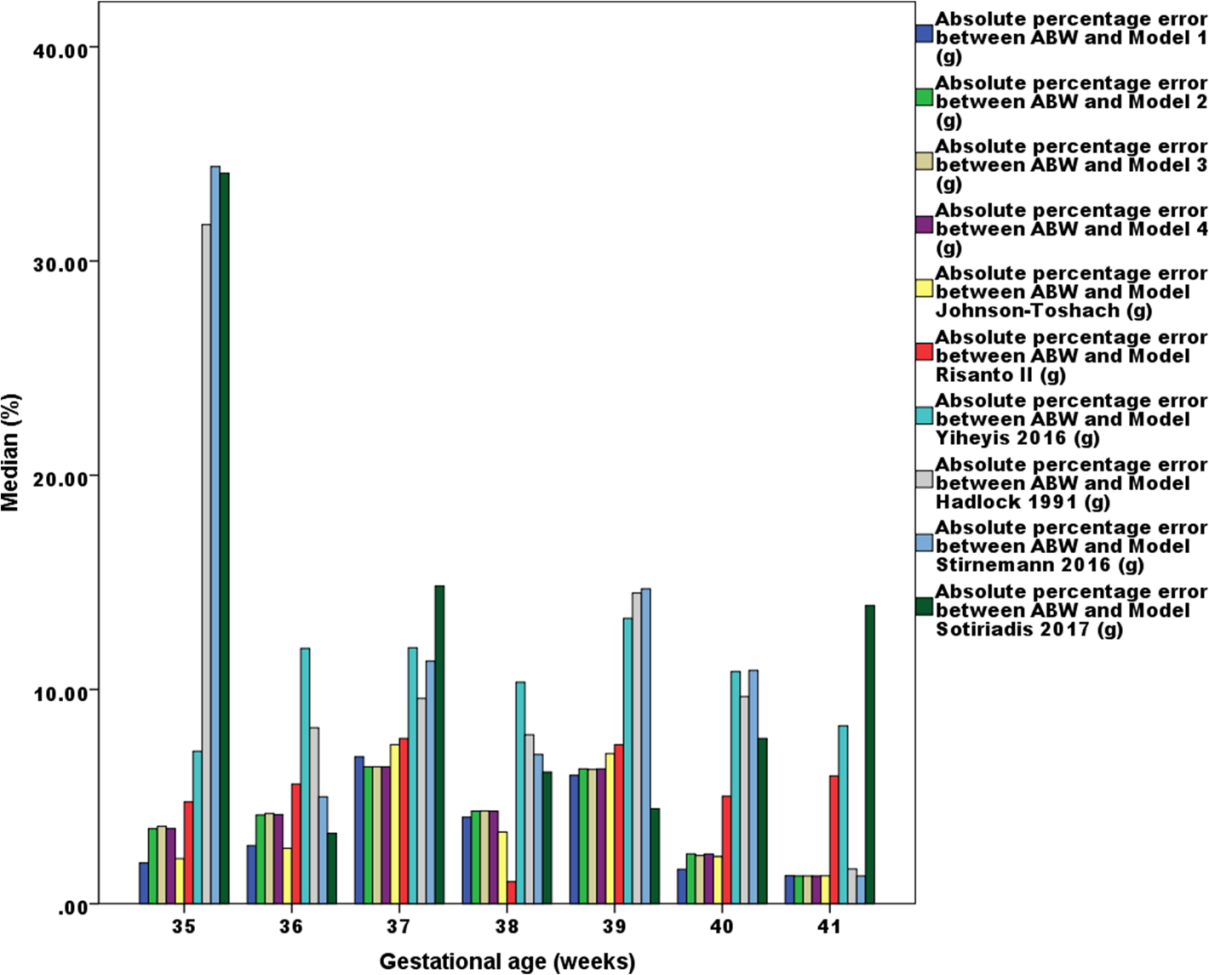
MEDAPEs comparison between the proposed and commonly used models ordered by GA

Table 3 shows that the mean prediction errors recorded for the proposed models are significantly smaller (between − 0.2 and 2.4 g) than those recorded for other existing models. Similarly, the MAPEs and MEDAPEs recorded for the proposed models are significantly smaller (between 5.0 and 5.1% and between 4.1 and 4.2%, respectively) than those recorded for other existing models. Therefore, we concluded that our four proposed models were capable to predict estimated foetal weight with less errors compare with the existing models between 35 and 41 weeks of pregnancy. The visualisation of these multiple comparisons can be seen in Fig. 3.

Furthermore, a two independent sample t-test (provided in Additional file 7: Table S7) was also used to investigate if there is a significant difference between the observed and estimated foetal weights based on the proposed models. The results show that there is no significant difference between the observed and estimated foetal weights based on the proposed models (*p*-value > 0.05).

### Assessing the prediction accuracy based on proportion falling between 10% of actual values

Table 3 presents the prediction ability of the proposed models, 11 existing clinical models (based on FH only), and 6 existing ultrasonic models (based on ultrasonic measurements of foetal HC and foetal AC as well as based on GA only). The table provides the total number of predictions falling within 10% of ABW.

Table 3 shows that 92% of the predicted values produced by our proposed Model (1) fall within the 10% of ABW compared with 89% for the Johnson-Toshach model and 84% of the Risanto models. However, Model (1) only uses FH to predict foetal weight, while the Johnson-Toshach model requires information on FH as well as FS. Therefore, we recommend that Model (1) be used instead of the Johnson-Toshach model.

Model (1) (based on FH only) is equally capable to estimate foetal weight as Models (2) and (4). These results imply that the inclusion of GA (which is not a biometric measurement of foetus) and estimated foetal AC do not have an impact on estimated foetal weight accuracy. Our results are in agreement with the previous study conducted by Huber (2014) [48].

Comparing the accuracy of Model (3) (based on FH and estimated foetal HC) and Model (4) (based on FH and estimated foetal AC) with the Hadlock 1984 model (based on ultrasonic measurements of foetal HC and foetal AC) [10], we concluded that both proposed Models (3) and (4) were significantly more capable in predicting foetal weight than the Hadlock model. Table 3 shows that the proportion of predicted birth weights falling within the 10% of ABW for Models (3) and (4) are more than double the proportion based on the Hadlock model.

## Discussion

Our study highlights that the use of combined maternal and estimated foetal biometric characteristics can provide a reliable estimate of delivery weights between 35 and 41 weeks of GA. This result confirms the previous study that shows a significant association between birth weight and characteristics of mother and foetus [1, 26].

Both clinical and estimates of ultrasonic predictors are used in our proposed models. Maternal FH measurement was selected as one of the clinical predictors as it is one of the most recommended and accessible predictors to estimate foetal weight and monitor foetal growth during pregnancy [3, 23, 49, 50]. Although the clinical approach using FH screening had reportedly low sensitivity for detecting intergrowth and birth weight abnormalities (ranged 16–45%) [51, 52], it is a simple and inexpensive clinical activity [29, 53], especially true in rural areas where ultrasound machines and skilled personnel are not always available. The utility of FH remains an important first level screening tool, widely used during routine ANC in both high and low income settings [29] even though it had high false-negative rates for small for GA [53].

In ultrasonic settings, foetal biometric characteristics monitored during pregnancy include HC, biparietal diameter (BPD), occipitofrontal diameter (OFD), AC, and femur length (FL). These characteristics are routinely measured by ultrasound every 5 weeks after the first initial dating scan (between 8 and 14 weeks’ gestation). The standard ranges for ultrasonic measurements are (14–18), (19–23), (24–28), (29–33), (34–38), and (39–42) weeks [54] or at least once every trimester of pregnancy, i.e. between weeks 10–14 (first trimester), 20–24 (second trimester), and 30–32 (third trimester) [55].

Assessment of foetal biometric characteristics during ANC is vital to ensuring normal foetal size and safe delivery. In the absence of ultrasound facility, particularly in low-resource primary health care settings, the measurements of these characteristics are not always accessible. Therefore, a reliable prediction of these characteristics during pregnancy would be a proxy of foetal biometrics and vitally improve the quality of ANC services in monitoring foetal inter growth assessment which currently remain low due to the database gaps [56–59].

Several ultrasonic formulas to estimate the foetal characteristics at different GA have been developed [30–32]. The foetal HC and foetal AC are widely recognised as the most influential predictors for predicting foetal weight [10, 11, 46, 60, 61]. Our results show that the best fit formulas to estimate these foetal characteristics at a given GA in our population were based on the Australian population [30].

To the best of our knowledge, in the majority of Indonesian primary health care centres where ultrasound facility is not accessible, none of the existing ultrasonic formulas were adopted to estimate foetal HC and foetal AC. Therefore, the formulas potentially can be deployed to fill in the database gaps on the inter growth process of foetus during pregnancy. Consequently, early informed intervention could be initiated to prevent abnormal growth and delivery weights.

Several techniques have been available to reduce collinearity, such as centering, multiplying variables by various constants (scaling), the use of orthogonal polynomials, and other transformations [62]. Currently, the use of automated machine learning, such as Genetic Algorithm rather than a conventional fractional polynomial approach has also been applied to model multiple biometric variables of foetus that are highly correlated [54].

In this paper, we used the best subset selection algorithm to prevent the inclusion of highly correlated variables and select the best subset of predictors to be included in the models. It has been emphasized that a formula for estimating foetal weight should be simple and straightforward to be used by doctors and midwives and be easily understood by patients [63]. This would improve the quality of communication, information, and education as part of routine ANC service in low-resource primary health care centres.

Based on our comparison analysis, the proposed Models (1), (2), (3), and (4) produced the least mean prediction errors (between − 0.2 and − 2.4 g), the MAPEs (between 5.01 and 5.10%), and the MEDAPEs (between 4.10 and 4.22%). The mean percentage prediction error (MPE) steadily tended towards zero as the time interval between the last scan and birth decreased [42]. Our MPEs were ranged between − 0.1 and − 0.3% in those born within 0 day (*n* = 38) which are lower than the previous research [42] reported by − 0.8% in those born within 1 day (*n* = 198).

Our proposed models were unbiased for predicting weight between 35 and 41 weeks of GA. In the group born within 0 day of the last measurements, the MAPEs were ranged between 5.0 and 5.10% with 89–92% of predicted weights falling within 10% of the true birth weights which are smaller than those reported in previous study [42]. This was particularly for Model (1) which was simply developed based on FH only.

The comparison between the proposed Model (1) and the widely used Johnson-Toshach model shows that Model (1) (developed based on the Indonesian data) was more accurate in predicting the estimated foetal weight than the Johnson-Toshach model (developed based on the United States data). Furthermore, the Johnson-Toshach model requires the knowledge of FS. The results presented in Table 3 also shows that the inclusion of FS in the model has not reduced the prediction errors in foetal weight estimations yet raise a subjectivity issue unless there is a standard protocol to determine FS with less error [20]. Therefore, we recommend the proposed Model (1) be deployed in Indonesia and other countries with similar health systems and challenges for weight prediction.

Our comparison study confirms that the proposed Models (3) (based on FH and estimates of foetal HC) and (4) (based on FH and estimates of foetal AC) perform better than the ultrasonic models: the Jordaan, the Weiner II, the Hadlocks, the Stirnemann, and the Sotiriadis models. The incorporation of estimated foetal HC or estimated foetal AC has increased *R*^2^ slightly (provided in Additional file 5: Table S5), but it did not improve the predicting accuracy (Table 3). However, access to these values will enable the practitioners to monitor foetal growth during pregnancy where advanced equipment, such as ultrasound, is not always available. Consequently, detecting foetal growth abnormality, such as small for GA, prematurity, intrauterine growth retardation, and LBW during pregnancy will be possible.

### Strengths and limitations

Our retrospective study has investigated the utilisation of some commonly used foetal weight prediction models in Indonesia. Particularly, the combination between maternal and estimated foetal biometric characteristics was proposed. The aim of this combination was whether it could improve the prediction accuracy of foetal weight at any given GA in the absence of ultrasound machines and trained ultra-sonographers.

The retrospective cohort study was undertaken to provide baseline data on the selected primary health care centre. It is possible that women have used different health services than that reviewed in this study. Although this may result in underestimation in data records, it is unlikely to impact on the validity of the analyses. This study also encountered limitations associated with the accuracy of the information recorded on the manual pregnancy register or inaccurate data transfer to the electronic database. However, monitoring and controlling the process of data transfer was conducted to reduce potential error. Further study should be conducted to assess the efficacy of the proposed models using prospective data [64].

The proposed prediction models are linear regressions. However, the authors have investigated non-linear models. The non-linear models did not improve the estimation accuracy. Therefore, complex models do not guarantee significant improvement in the prediction accuracy. Furthermore, due to the fact that the objective of the study is to provide simple yet reliable foetal weight estimating models for low-resource areas, we are recommending the proposed models. We believe that the findings can be applied in other low-resource settings to improve ANC services.

## Conclusion

This research has developed models to predict the estimated foetal weight at varying gestational age where ultrasound facilities do not exist. Since birth weight is one of the most important indicators of neonatal survival, a reliable estimate of foetal weight at different stages of pregnancy would facilitate the intervention plan for medical practitioners to prevent the risk of abnormal delivery weights. Further, the models will lead to the development of foetal inter growth charts, which are currently unavailable in the Indonesian primary health care systems.

## Additional files

Additional file 1: **Table S1.** Existing ultrasonic formulas to estimate foetal HC and AC based on GA. Table S1 consists of the existing ultrasonic formulas to estimate foetal head circumference (HC) and foetal abdominal circumference (AC) which were developed based on the Australian foetal biometry data (measured between 11 and 41 weeks), the UK foetal biometry data (measured between 13 and 42 weeks), and the international foetal biometry data (measured between 14 and 42 weeks or until birth) [29–31]. (PDF 167 kb)

Additional file 2: **Table S2.** Intraclass correlation coefficient analysis of the existing ultrasonic formulas in predicting foetal biometrics. Table S2 shows a reliability analysis using intraclass correlation coefficient (ICC) to assess the consistency of the ultrasonic formulas for Indonesian population. The obtained ICC values were computed by single-rating, consistency, and two-way random effects models for the foetal biometrics with three raters (different ultrasonic formulas) across 127 subjects (pregnant women). (PDF 95 kb)

Additional file 3: **Table S3.** Interclass correlation coefficient analysis for predicting foetal biometrics. Table S3 describes interclass (Pearson) correlation coefficient to assess whether there is a significant relationship between the predicted foetal biometrics and the neonatal measurements recorded at delivery time. (PDF 108 kb)

Additional file 4: **Table S4.** Correlation coefficient of the potentially clinical predictors of foetal weight estimation. Table S4 presents the investigation of correlations between the potential predictors of foetal weight estimation based on 127 data. (PDF 91 kb)

Additional file 5: **Table S5.** Models recommended by the best subset selection algorithm together with corresponding analysis of variance information. Table S5 summarises the models developed based on the recorded estimated foetal weight (EFW_r_) using the best subset selection algorithm. These models were based on one, two, and three independent variables. The table also lists their corresponding *R*^2^, Mallows C_p_, *S*, and *VIF* statistics. (PDF 171 kb)

Additional file 6: **Table S6.** List of the proposed and existing models based on clinical and ultrasonic variables. Table S6 lists the proposed models and the existing clinical and ultrasonic models for estimating foetal weight. (PDF 381 kb)

Additional file 7: **Table S7.** Two independent sample t-tests between ABW, EFW_r_, and EFW_p_. Table S7 provides a two independent sample t-test to investigate if there is a significant difference between the observed or actual values of birth weight (ABW), recorded foetal weight estimation (EFW_r_), and estimated foetal weights based on the proposed model (EFW_p_). (PDF 157 kb)

## Abbreviations

ABW: Actual birth weight
ABW_*i*_: The *i*^th^ actual values of birth weight
AC: Abdominal circumference
ANC: Antenatal care
ANOVA: Analysis of variance
BPD: Biparietal diameter
CIE: Communication, information, and education
EFW_p_: Estimated foetal weight based on the proposed model
EFW_r_: Recorded foetal weight estimation
EFW_pi_: The *i*^th^ predicted values of foetal weight based on the proposed models
FH: Fundal height
FL: Femur length
FS: Foetal head engagement/foetal station
GA: Gestational age
HC: Head circumference
ICC: Intraclass correlation coefficient
LBW: Low birth weight
MAPE: Mean absolute percentage prediction error
MEDAPE: Median absolute percentage prediction error
MPE: Mean percentage prediction error
OFD: Occipitofrontal diameter
OLS: Ordinary least square
*r*: Correlation coefficient
*R*^2^: Coefficient of determination
*S*: Standard deviation
*VIF*: Variance inflation factor

## References

[1] Njim T, Atashili J, Mbu R, Choukem S-P (2015). Low birth weight in a sub-urban area of Cameroon: an analysis of the clinical cut-off, incidence, predictors and complications. BMC Pregnancy Childbirth.

[2] Gibson KS, Waters TP, Gunzler DD, Catalano PM (2015). A retrospective cohort study of factors relating to the longitudinal change in birth weight. BMC Pregnancy Childbirth.

[3] Parvin Z, Shafiuddin S, Uddin MA, Begum F (2013). Symphysio fundal height (SFH) measurement as a predictor of birth weight. Faridpur Med Coll J.

[4] Lalys L, Pineau J-C, Guihard-Costa A-M (2010). Small and large foetuses: identification and estimation of foetal weight at delivery from third-trimester ultrasound data. Early Hum Dev.

[5] Sharma SR, Giri S, Timalsina U, Bhandari SS, Basyal B, Wagle K, Shrestha L (2015). Low birth weight at term and its determinants in a tertiary hospital of Nepal: a case-control study. PLoS One.

[6] Willocks J, Donald I, Duggan T, Day N (1964). Foetal cephalometry by ultrasound. BJOG Int J Obstet Gynaecol.

[7] Robson SC, Gallivan S, Walkinshaw SA, Vaughan J, Rodeck CH (1993). Ultrasonic estimation of fetal weight: use of targeted formulas in small for gestational age fetuses. Obstet Gynecol.

[8] Spinnato JA, Allen RD, Mendenhall HW (1988). Birth weight prediction from remote ultrasound examination. Obstet Gynecol.

[9] Shepard M, Richards V, Berkowitz R, Warsof S, Hobbins J (1982). An evaluation of two equations for predicting fetal weight by ultrasound. Am J Obstet Gynecol.

[10] Hadlock F, Harrist R, Carpenter R, Deter R, Park S (1984). Sonographic estimation of fetal weight. The value of femur length in addition to head and abdomen measurements. Radiology.

[11] Hadlock FP, Harrist R, Sharman RS, Deter RL, Park SK (1985). Estimation of fetal weight with the use of head, body, and femur measurements—a prospective study. Am J Obstet Gynecol.

[12] Campbell S, Wilkin D (1975). Ultrasonic measurement of fetal abdomen circumference in the estimation of fetal weight. BJOG Int J Obstet Gynaecol.

[13] Combs CA, Jaekle RK, Rosenn B, Pope M, Miodovnik M, Siddiqi TA (1993). Sonographic estimation of fetal weight based on a model of fetal volume. Obstet Gynecol.

[14] Johnson R, Toshach C (1954). Estimation of fetal weight using longitudinal mensuration. Am J Obstet Gynecol.

[15] Johnson RW (1957). Calculations in estimating fetal weight. Am J Obstet Gynecol.

[16] Niswander KR, Capraro VJ, Van Coevering RJ (1970). Estimation of birth weight by quantified external uterine measurements. Obstet Gynecol.

[17] Farid SW (1999). Child weight prediction based on the modification of the Niswander formula (Taksasi berat badan anak berdasarkan modifikasi rumus Niswander). Majalah Obstetri dan Ginekologi Indonesia.

[18] Buchmann E, Tlale K (2009). A simple clinical formula for predicting fetal weight in labour at term: derivation and validation. SAMJ.

[19] Mongelli M, Gardosi J (2004). Estimation of fetal weight by symphysis–fundus height measurement. Int J Gynecol Obstet.

[20] Bothner B, Gulmezoglu A, Hofmeyr G (2000). Symphysis fundus height measurements during labour: a prospective, descriptive study. Afr J Reprod Health.

[21] Dare F, Ademowore A, Ifaturoti O, Nganwuchu A (1990). The value of symphysio-fundal height/abdominal girth measurements in predicting fetal weight. Int J Gynecol Obstet.

[22] Siswosudarmo H (1995). Detection of low birth weight babies at term pregnancy with fundal height measurements (Deteksi bayi berat lahir rendah pada kehamilan aterm dengan pengukuran tinggi fundus). Berkala Epidemiologi Klinik Biostatika Indonesia.

[23] Siswosudarmo R, Titisari I. Developing a new formula for estimating birth weight at term pregnancy. Jurnal Kesehatan Reproduksi. 2014;1(2):145–149.

[24] Gayatri D, Afiyanti Y (2006). Validation of fetal weight estimation formula (TBJ) for prediction of birth weight based on uterine fundal height of pregnant women (Validasi rumus taksiran berat janin (TBJ) untuk prediksi berat badan lahir berdasarkan tinggi fundus uterus ibu hamil). Jurnal Keperawatan Indonesia.

[25] Santjaka HI, Handayani R (2011). Study of the accuracy of fetal weight estimates based on statistics and fundal height (Studi ketepatan taksiran berat janin berdasarkan statistik dan tinggi fundus uteri). Jurnal Bidan Prada.

[26] Kiserud T, Piaggio G, Carroli G, Widmer M, Carvalho J, Jensen LN, Giordano D, Cecatti JG, Aleem HA, Talegawkar SA (2017). The World Health Organization fetal growth charts: a multinational longitudinal study of ultrasound biometric measurements and estimated fetal weight. PLoS Med.

[27] Anggraini D, Abdollahian M, Marion K (2015). Review of low birth weight prediction models in Indonesia. Int J Adv Sci Eng Technol.

[28] Abdollahian M, Ahmad S, Huda S, Nuryani S, Anggraini D (2012). Investigating the relationship between neonatal mortality rate and mother’s characteristics. Proceedings of the International Conference on Information and Knowledge Engineering (IKE); The Steering Committee of the World Congress in Computer Science, Computer Engineering, and Applied Computing (WorldComp); CSREA Press.

[29] Papageorghiou AT, Ohuma EO, Gravett MG, Hirst J, da Silveira MF, Lambert A, Carvalho M, Jaffer YA, Altman DG, Noble JA (2016). International standards for symphysis-fundal height based on serial measurements from the fetal growth longitudinal study of the INTERGROWTH-21st project: prospective cohort study in eight countries. BMJ.

[30] Westerway SC, Davison A, Cowell S (2000). Ultrasonic fetal measurements: new Australian standards for the new millennium. Aust N Z J Obstet Gynaecol.

[31] Loughna P, Chitty L, Evans T, Chudleigh T (2009). Fetal size and dating: charts recommended for clinical obstetric practice. Ultrasound.

[32] Papageorghiou AT, Ohuma EO, Altman DG, Todros T, Ismail LC, Lambert A, Jaffer YA, Bertino E, Gravett MG, Purwar M (2014). International standards for fetal growth based on serial ultrasound measurements: the fetal growth longitudinal study of the INTERGROWTH-21 st project. Lancet.

[33] Achadi E, Jones G (2014). Health sector review: maternal, neonatal, and child health. Jakarta: Ministry of National Development Planning/Bappenas, Republic of Indonesia.

[34] UNICEF-Indonesia (2012). Issues briefs: maternal and child health. UNICEF Indonesia.

[35] MoH (2013). Indonesia health profile (Profil kesehatan Indonesia) 2012. Jakarta: Ministry of Health, Republic of Indonesia.

[36] Bellio R, Ventura L (2005). An introduction to robust estimation with R functions. Proceedings of 1st International Work.

[37] Fox J, Weisberg S (2010). An R companion to applied regression: Sage.

[38] Renaud O, Victoria-Feser M-P (2010). A robust coefficient of determination for regression. J Statist Plann Inference.

[39] Faraway JJ (2002). Practical regression and ANOVA using R. University of Bath.

[40] Landers R (2015). Computing Intraclass Correlations (ICC) as estimates of interrater reliability in SPSS. The Winnower 2: e143518. 81744.

[41] Koo TK, Li MY (2016). A guideline of selecting and reporting intraclass correlation coefficients for reliability research. J Chiropr Med.

[42] Stirnemann J, Villar J, Salomon L, Ohuma E, Ruyan P, Altman D, Nosten F, Craik R, Munim S, Cheikh Ismail L (2017). International estimated fetal weight standards of the INTERGROWTH-21st project. Ultrasound Obstet Gynecol.

[43] Levenbach H (2015). Training EDC: The Myth of the MAPE and how to avoid it.

[44] Anggraini D, Abdollahian M, Marion K (2016). Accuracy assessment on prediction models for fetal weight based on maternal fundal height. Information technology: new generations.

[45] Yiheyis A, Alemseged F, Segni H (2016). Johnson’s formula for predicting birth weight in pregnant mothers at Jimma University Teaching Hospital, South West Ethiopia. Med J Obstet Gynecol.

[46] Jordaan HV (1983). Estimation of fetal weight by ultrasound. J Clin Ultrasound.

[47] Weiner Z, Ben-Shlomo I, Beck-Fruchter R, Goldberg Y, Shalev E (2002). Clinical and ultrasonographic weight estimation in large for gestational age fetus. Eur J Obstet Gynecol Reprod Biol.

[48] Huber C, Zdanowicz JA, Mueller M, Surbek D (2014). Factors influencing the accuracy of fetal weight estimation with a focus on preterm birth at the limit of viability: a systematic literature review. Fetal Diagn Ther.

[49] Morse K, Williams A, Gardosi J (2009). Fetal growth screening by fundal height measurement. Best Pract Res Clin Obstet Gynaecol.

[50] Titisari HI, Siswosudarmo R (2013). Risanto’s formulas is more accurate in determining estimated fetal weight based on maternal fundal height. Indones J Obstet Gynecol.

[51] Sparks TN, Cheng YW, McLaughlin B, Esakoff TF, Caughey AB (2011). Fundal height: a useful screening tool for fetal growth?. J Matern Fetal Neonatal Med.

[52] Curti A, Zanello M, De Maggio I, Moro E, Simonazzi G, Rizzo N, Farina A (2014). Multivariable evaluation of term birth weight: a comparison between ultrasound biometry and symphysis-fundal height. J Matern Fetal Neonatal Med.

[53] Pay ASD, Wiik J, Backe B, Jacobsson B, Strandell A, Klovning A (2015). Symphysis-fundus height measurement to predict small-for-gestational-age status at birth: a systematic review. BMC Pregnancy Childbirth.

[54] Papageorghiou AT, Kemp B, Stones W, Ohuma EO, Kennedy SH, Purwar M, Salomon LJ, Altman DG, Noble JA, Bertino E (2016). Ultrasound-based gestational-age estimation in late pregnancy. Ultrasound Obstet Gynecol.

[55] Postoev VA, Grjibovski AM, Nieboer E, Odland JØ (2015). Changes in detection of birth defects and perinatal mortality after introduction of prenatal ultrasound screening in the Kola Peninsula (North-West Russia): combination of two birth registries. BMC Pregnancy Childbirth.

[56] Gardosi J (2011). Fetal growth standards: individual and global perspectives. Lancet.

[57] Gardosi Jason (2012). Customised assessment of fetal growth potential: implications for perinatal care. Archives of Disease in Childhood - Fetal and Neonatal Edition.

[58] Moxon SG, Ruysen H, Kerber KJ, Amouzou A, Fournier S, Grove J, Moran AC, Vaz LM, Blencowe H, Conroy N (2015). Count every newborn: a measurement improvement roadmap for coverage data. BMC Pregnancy Childbirth.

[59] Kerber KJ, Mathai M, Lewis G, Flenady V, Erwich JJH, Segun T, Aliganyira P, Abdelmegeid A, Allanson E, Roos N (2015). Counting every stillbirth and neonatal death through mortality audit to improve quality of care for every pregnant woman and her baby. BMC Pregnancy Childbirth.

[60] Abele H, Hoopmann M, Wagner N, Hahn M, Wallwiener D, Kagan KO (2010). Accuracy of sonographic fetal weight estimation of fetuses with a birth weight of 1500g or less. Eur J Obstet Gynecol Reprod Biol.

[61] Dudley N (2005). A systematic review of the ultrasound estimation of fetal weight. Ultrasound Obstet Gynecol.

[62] Kleinbaum D, Kupper L, Nizam A, Rosenberg E (2013). Applied regression analysis and other multivariable methods: Nelson Education.

[63] Salomon LJ, Bernard JP, Ville Y (2007). Estimation of fetal weight: reference range at 20–36 weeks’ gestation and comparison with actual birth-weight reference range. Ultrasound Obstet Gynecol.

[64] Asiki G, Baisley K, Newton R, Marions L, Seeley J, Kamali A, Smedman L (2015). Adverse pregnancy outcomes in rural Uganda (1996–2013): trends and associated factors from serial cross sectional surveys. BMC Pregnancy Childbirth.

[65] Gayatri D, Afiyanti Y (2004). Comparison of some formulas for predicting birth weight based on height measurement of fundus uteri (Perbandingan beberapa rumus untuk memprediksi berat badan lahir berdasrkan pengukuran tinggi fundus uteri). Jurnal Keperawatan Indonesia.

[66] Rusdy RS, Yasmin FA, Putri LA, Oktrian O, Pusponegoro A (2014). Comparison of Johnson-Tohsach formula with South Africa formula in determining fetal weight estimation at Puskesmas Kecamatan Pasar Rebo, East Jakarta (Perbandingan rumus Johnson-Tohsach dengan rumus South Africa dalam menentukan taksiran berat janin di Puskesmas Kecamatan Pasar Rebo, Jakarta Timur). eJurnal Kedokteran Indonesia.

[67] Hadlock FP, Harrist RB, Martinez-Poyer J (1991). In utero analysis of fetal growth: a sonographic weight standard. Radiology.

[68] Sotiriadis A, Eleftheriades M, Papadopoulos V, Sarafidis K, Pervanidou P, Assimakopoulos E (2018). Divergence of estimated fetal weight and birth weight in singleton fetuses. J Matern Fetal Neonatal Med.

