# Supplementary material for: Foetal weight prediction models at a given gestational age in the absence of ultrasound facilities: application in Indonesia
Source: BMC Pregnancy Childbirth. 2018 Nov 6;18:436. doi: 10.1186/s12884-018-2047-z (PMC6219176; doi:10.1186/s12884-018-2047-z)
Supplement: Supplementary file 1 — Table S1. Existing ultrasonic formulas to estimate foetal HC and AC based on GA. Table S1 consists of the existing ultrasonic formulas to estimate foetal head circumference (HC) and foetal abdominal circumference (AC) which were developed based on the Australian foetal biometry data (measured between 11 and 41 weeks), the UK foetal biometry data (measured between 13 and 42 weeks), and the international foetal biometry data (measured between 14 and 42 weeks or until birth) [29–31]. (PDF 167 kb) [file 12884_2018_2047_MOESM1_ESM.pdf]

Table S1 Existing ultrasonic formulas to estimate foetal HC and AC based on GA

**Ultrasonic formulas**	**HC**	**AC**
Australian standard [1]	HC = -127.91 + 18.494 GA – 0.1699 GA^2	AC = -90.946 + 13.204 GA – 0.0469 GA^2
UK standard [2]	HC = -109.7 + 15.16 GA – 0.002388 GA^3	AC = -85.84 + 11.92 GA – 0.0007902 GA^3
International standard [3]	HC = -28.2849 + 1.69267 × GA^2 – 0.397485 × GA^2 × log (GA)	AC = -81.3243 + 11.6772 × GA – 0.000561865 × GA^3

Where: HC = foetal head circumference (mm); AC = foetal abdominal circumference (mm); GA = gestational age (weeks)

References:

1. Westerway SC, Davison A, Cowell S. Ultrasonic fetal measurements: new Australian standards for the new millennium. Australian and New Zealand Journal of Obstetrics and Gynaecology 2000; 40(3):297-302.
2. Loughna P, Chitty L, Evans T, Chudleigh T. Fetal size and dating: charts recommended for clinical obstetric practice. Ultrasound 2009; 17(3):160-166.
3. Papageorghiou AT, Ohuma EO, Altman DG, Todros T, Ismail LC, Lambert A, Jaffer YA, Bertino E, Gravett MG, Purwar M. International standards for fetal growth based on serial ultrasound measurements: the Fetal Growth Longitudinal Study of the INTERGROWTH-21 st Project. The Lancet 2014; 384(9946):869-879.
