# Supplementary material for: Foetal weight prediction models at a given gestational age in the absence of ultrasound facilities: application in Indonesia
Source: BMC Pregnancy Childbirth. 2018 Nov 6;18:436. doi: 10.1186/s12884-018-2047-z (PMC6219176; doi:10.1186/s12884-018-2047-z)
Supplement: Supplementary file 2 — Table S2. Intraclass correlation coefficient analysis of the existing ultrasonic formulas in predicting foetal biometrics. Table S2 shows a reliability analysis using intraclass correlation coefficient (ICC) to assess the consistency of the ultrasonic formulas for Indonesian population. The obtained ICC values were computed by single-rating, consistency, and two-way random effects models for the foetal biometrics with three raters (different ultrasonic formulas) across 127 subjects (pregnant women). (PDF 95 kb) [file 12884_2018_2047_MOESM2_ESM.pdf]

Table S2 Intraclass correlation coefficient analysis of the existing ultrasonic formulas in predicting foetal biometrics

**Foetal biometrics**	**Intraclass correlation coefficient (single measure)**	**95% Confidence interval**		**F test with true value 0**			
		**Lower bound**	**Upper bound**	**Value**	**df1**	**df2**	**Sig**
HC	0.957	0.943	0.968	67.704	126	252	.000
AC	0.996	0.995	0.997	738.785	126	252	.000
