# Supplementary material for: Foetal weight prediction models at a given gestational age in the absence of ultrasound facilities: application in Indonesia
Source: BMC Pregnancy Childbirth. 2018 Nov 6;18:436. doi: 10.1186/s12884-018-2047-z (PMC6219176; doi:10.1186/s12884-018-2047-z)
Supplement: Supplementary file 3 — Table S3. Interclass correlation coefficient analysis for predicting foetal biometrics. Table S3 describes interclass (Pearson) correlation coefficient to assess whether there is a significant relationship between the predicted foetal biometrics and the neonatal measurements recorded at delivery time. (PDF 108 kb) [file 12884_2018_2047_MOESM3_ESM.pdf]

Table S3 Interclass correlation efficient analysis for predicting foetal biometrics

Reference	Variables	Complete samples	Pearson correlation coefficient (r)	Number of estimates within 10% of actual values	
				n	%
Australian standard [1]	Estimated HC	127	0.212*	125	98
	Estimated AC		0.078	112	88
UK standard [2]	Estimated HC		0.205*	125	98
	Estimated AC		0.076	104	82
International standard [3]	Estimated HC		0.191*	125	98
	Estimated AC		0.077	107	84

*The p-value of less than 0.05 confirms significant correlation

References:

1. Westerway SC, Davison A, Cowell S. Ultrasonic fetal measurements: new Australian standards for the new millennium. Australian and New Zealand Journal of Obstetrics and Gynaecology 2000; 40(3):297-302.
2. Loughna P, Chitty L, Evans T, Chudleigh T. Fetal size and dating: charts recommended for clinical obstetric practice. Ultrasound 2009; 17(3):160-166.
3. Papageorghiou AT, Ohuma EO, Altman DG, Todros T, Ismail LC, Lambert A, Jaffer YA, Bertino E, Gravett MG, Purwar M. International standards for fetal growth based on serial ultrasound measurements: the Fetal Growth Longitudinal Study of the INTERGROWTH-21 st Project. The Lancet 2014; 384(9946):869-879.
