# Supplementary material for: Foetal weight prediction models at a given gestational age in the absence of ultrasound facilities: application in Indonesia
Source: BMC Pregnancy Childbirth. 2018 Nov 6;18:436. doi: 10.1186/s12884-018-2047-z (PMC6219176; doi:10.1186/s12884-018-2047-z)
Supplement: Supplementary file 4 — Table S4. Correlation coefficient of the potentially clinical predictors of foetal weight estimation. Table S4 presents the investigation of correlations between the potential predictors of foetal weight estimation based on 127 data. (PDF 91 kb) [file 12884_2018_2047_MOESM4_ESM.pdf]

Table S4 Correlation coefficient of the potentially clinical predictors of foetal weight estimation

	**GA**	**FH**	**EFW_r**
**FH**	0.100		
**EFW_r**	0.046	0.952*	
**ABW**	0.030	0.795*	0.825*

*The p-value of less than 0.05 confirms significant correlation
