# Supplementary material for: Foetal weight prediction models at a given gestational age in the absence of ultrasound facilities: application in Indonesia
Source: BMC Pregnancy Childbirth. 2018 Nov 6;18:436. doi: 10.1186/s12884-018-2047-z (PMC6219176; doi:10.1186/s12884-018-2047-z)
Supplement: Supplementary file 5 — Table S5. Models recommended by the best subset selection algorithm together with corresponding analysis of variance information. Table S5 summarises the models developed based on the recorded estimated foetal weight (EFWr) using the best subset selection algorithm. These models were based on one, two, and three independent variables. The table also lists their corresponding R2, Mallows Cp, S, and VIF statistics. (PDF 171 kb) [file 12884_2018_2047_MOESM5_ESM.pdf]

Table S5 Models recommended by the best subset selection algorithm together with corresponding analysis of variance information

Model	Number of variables	R^2 (%)	R^2 (adj) (%)	R^2 (pred) (%)	Mallows Cp	S (g)	GA (weeks)	FH	Estimated foetal HC (cm)	Estimated foetal AC (cm)
(1)	1	88.3	88.2	87.9	3.4	123.90		√		
(2)	2	88.8	88.5	87.6	3.6	122.27	√ (VIF^+ = 1.01)	√ (VIF^+ = 1.01)		
(3)	2	88.8	88.5	87.6	2.0	122.22		√ (VIF^+ = 1.01)	√ (VIF^+ = 1.01)	
(4)	2	88.8	88.5	87.6	2.1	122.26		√ (VIF^+ = 1.01)		√ (VIF^+ = 1.01)
(5)	3	88.8	88.4	82.5	4.0	122.92		√ (VIF^+ = 1.01)	√ (VIF^+ = 194.80)	√ (VIF^+ = 194.88)

$^+$Mallows Cp close to p (the number of explanatory variables) indicates a good fit and VIF > 10 indicates the presence of multicollinearity.
