# Supplementary material for: Foetal weight prediction models at a given gestational age in the absence of ultrasound facilities: application in Indonesia
Source: BMC Pregnancy Childbirth. 2018 Nov 6;18:436. doi: 10.1186/s12884-018-2047-z (PMC6219176; doi:10.1186/s12884-018-2047-z)
Supplement: Supplementary file 6 — Table S6. List of the proposed and existing models based on clinical and ultrasonic variables. Table S6 lists the proposed models and the existing clinical and ultrasonic models for estimating foetal weight. (PDF 381 kb) [file 12884_2018_2047_MOESM6_ESM.pdf]

Table S6 List of the proposed and existing models based on clinical and ultrasonic variables

Authors	Variables	Formulas
**Our recommended models**		
Dewi, Mali & Kaye	FH	**Model (1):** **EFW (g) = 150.293 x FH – 1538.331**
	FH, GA	**Model (2):** **EFW (g) = 151.19 x FH – 15.75 x GA – 959**
	FH, EHC	**Model (3):** **EFW (g) = 151.170 x FH – 2.806 x EHC – 634.288**
	FH, EAC	**Model (4):** **EFW (g) = 151.1889 x FH – 1.6339 x EAC – 996.7524**
**Existing clinical models**		
Johnson-Toshach (1954, 1957) [4, 5]	FH, n	**Johnson and Toshach's formula:** **EFW (g) = [FH – n] x 155** [6]. "If a pregnant woman weighs more than 91 kg, 1 cm is subtracted from the fundal height" [7].
Siswosudarmo, H (1995) cited in [8]	FH	**Risanto I Formula:** **EFW (g) = 126.7 x FH – 931.5**
Siswosudarmo, R & Titisari 2014 [9]	FH	**Risanto II Formula:** **EFW (g) = 125 x FH – 880**
Niswander, Capraro & Van Coevering (1970) cited in [10]	FH	**Niswander formula:** **EFW (g) = ((FH – 13)/3) x 453.6**
Farid & Sukarya (1999) cited in [10]	FH	**Modified Niswander formula:** **EFW (g) = (1.12 x FH – 7.7) x 100**
Mhaskar (2003) cited in [10]	FH	**Mhaskar formula:** **EFW (kg) = 0.18 x FH – 2.89**
Gayatri-Afiyanti Formula (2006) [11]	FH	**Gayatri-Afiyanti formula:** **EFW (g) = (FH – 4) x 100**
Buchmann and Tlale (2009) [12, 13]	FH	**Buchmann-Tlale Formula:** **EFW (g) = 100 x ([FH] – 5)**
Santjaka & Handayani 2011 [14]	FH	**Santjaka-Handayani Formula:** **EFW (kg) = 1.876 + 0.119 x FH**
Mongelli & Gardosi 2004 [15]	FH	**Mongelli-Gardosi Formula:** **ln EFW (g) = 10.6857 – 100.25 / FH**
Yiheyis, Alemsegned & Segni 2016 [16]	FH	**Yiheyis, Alemsegned & Segni formula:** **EFW (g) = 2600 + 155 x (FH – 30)**
**Existing ultrasonic models**		
Jordaan (1983)[17]	HC, AC	**Jordaan formula:** **$\log_{10}$EFW (g) = 0.9119 + 0.0488 x HC + 0.0824 x AC – 0.001599 x (AC x HC)**
Weiner II (1985) in [18]	HC, AC	**Weiner II formula:** **EFW (g) = 10^(1.6575 + 0.04035 x HC + 0.01285 x AC)**

Hadlock (1984) [19]	HC, AC	**Hadlock formula:** <br>$\log_{10}EFW\ (g) = 1.182 + 0.0273 \times HC + 0.07057 \times AC - 0.00063 \times AC^2 - 0.0002184 \times (HC \times AC)$
Hadlock (1991) [20]	GA	**Hadlock formula:** <br>$\log\ (EFW)\ (g) = 0.578 + 0.332 \times GA - 0.00354 \times GA^2$ <br>The log function designates the natural logarithm
Stirnemann et. al (2016) [21]	HC, AC	**International EFW standard formula:** <br>$\log\ (EFW)\ (g) = 5.084820 - 54.06633 \times (AC/100)^3 - 95.80076 \times (AC/100)^3 \times \log\ (AC/100) + 3.136370 \times (HC/100)$ <br>The log function designates the natural logarithm.
Sotiriadis et. al (2017) [22]	GA	**Sotiriadis et. al formula:** <br>$\log_{10}EFW\ (g) = -\ 0.204661 + 0.173510 \times GA - 0.002016 \times GA^2$

*Where: EFW= estimated foetal weight, FH = fundal height (cm), HC = foetal head circumference (cm); AC = foetal abdominal circumference (cm); GA = gestational age (weeks); EHC = estimated foetal head circumference (cm); EAC = estimated foetal abdominal circumference (cm); n = 13 if vertex is **above** ischial spine, or when the station is minus or foetal head is unengaged, n = 12 if vertex is **at** ischial spine or when the station is 0, n = 11 if vertex is **below** ischial spine or when the station is plus or foetal head is engaged.

References:

4. Johnson R, Toshach C. Estimation of fetal weight using longitudinal mensuration. American Journal of Obstetrics and Gynecology 1954; 68(3):891-896.
5. Johnson RW. Calculations in estimating fetal weight. American Journal of Obstetrics & Gynecology 1957; 74(4):929.
6. Chithra SC, Kumari LK, Sangeereni M. Comparative study of fetal weight estimation using Hadlock's and Johnson's formula and its correlation with actual birth weight. International Journal of Scientific Study 2014; 2(7):163-170.
7. Khani S, Ahmad-Shirvani M, Mohseni-Bandpei MA, Mohammadpour-Tahmtan RA. Comparison of abdominal palpation, Johnson’s technique and ultrasound in the estimation of fetal weight in Northern Iran. Midwifery 2011; 27(1):99-103.
8. Titisari HI, Siswosudarmo R. Risanto’s formulas is more accurate in determining estimated fetal weight based on maternal fundal height. Indonesian Journal of Obstetrics and Gynecology 2013; 1(3):149-151.
9. Siswosudarmo R, Titisari I. Developing a new formula for estimating birth weight at term pregnancy. Jurnal Kesehatan Reproduksi 2014; 1(2).
10. Gayatri D, Afiyanti Y. Comparison of some formulas for predicting birth weight based on height measurement of fundus uteri (Perbandingan beberapa rumus untuk memprediksi berat badan lahir berdasrkan pengukuran tinggi fundus uteri). Jurnal Keperawatan Indonesia 2004; 8(1):18-22.
11. Gayatri D, Afiyanti Y. Validation of fetal weight estimation formula (TBJ) for prediction of birth weight based on uterine fundal height of pregnant women (Validasi rumus taksiran berat janin (TBJ) untuk prediksi berat badan lahir berdasarkan tinggi fundus uterus ibu hamil). Jurnal Keperawatan Indonesia 2006; 10(1):24-29.
12. Buchmann E, Tlale K. A simple clinical formula for predicting fetal weight in labour at term: derivation and validation. SAMJ: South African Medical Journal 2009; 99(6):457-460.

13. Rusdy RS, Yasmin FA, Putri LA, Oktrian O, Pusponegoro A. Comparison of Johnson-Tohsach formula with South Africa formula in determining fetal weight estimation at Puskesmas Kecamatan Pasar Rebo, East Jakarta (Perbandingan rumus Johnson-Tohsach dengan rumus South Africa dalam menentukan taksiran berat janin di Puskesmas Kecamatan Pasar Rebo, Jakarta Timur). eJurnal Kedokteran Indonesia 2014; 2(1):33-36.
14. Santjaka HI, Handayani R. Study of the accuracy of fetal weight estimates based on statistics and fundal height (Studi ketepatan taksiran berat janin berdasarkan statistik dan tinggi fundus uteri). Jurnal Bidan Prada 2011; 2(01):21-34.
15. Mongelli M, Gardosi J. Estimation of fetal weight by symphysis–fundus height measurement. International Journal of Gynecology and Obstetrics 2004; 85(1):50-51.
16. Yiheyis A, Alemseged F, Segni H. Johnson's formula for predicting birth weight in pregnant mothers at Jimma University Teaching Hospital, South West Ethiopia. Medical Journal of Obstetrics and Gynecology 2016; 4(3):1087-1093.
17. Jordaan HV. Estimation of fetal weight by ultrasound. Journal of Clinical Ultrasound 1983; 11(2):59-66.
18. Abele H, Hoopmann M, Wagner N, Hahn M, Wallwiener D, Kagan KO. Accuracy of sonographic fetal weight estimation of fetuses with a birth weight of 1500g or less. European Journal of Obstetrics & Gynecology and Reproductive Biology 2010; 153(2):131-137.
19. Hadlock F, Harrist R, Carpenter R, Deter R, Park S. Sonographic estimation of fetal weight. The value of femur length in addition to head and abdomen measurements. Radiology 1984; 150(2):535-540.
20. Hadlock FP, Harrist RB, Martinez-Poyer J. In utero analysis of fetal growth: a sonographic weight standard. Radiology 1991; 181(1):129-133.
21. Stirnemann J, Villar J, Salomon L, Ohuma E, Ruyan P, Altman D, Nosten F, Craik R, Munim S, Cheikh Ismail L. International estimated fetal weight standards of the INTERGROWTH-21st Project. Ultrasound in obstetrics & gynecology 2017; 49(4):478-486.
22. Sotiriadis A, Eleftheriades M, Papadopoulos V, Sarafidis K, Pervanidou P, Assimakopoulos E. Divergence of estimated fetal weight and birth weight in singleton fetuses. The Journal of Maternal-Fetal & Neonatal Medicine 2018; 31(6):761-769.
