# Supplementary material for: Foetal weight prediction models at a given gestational age in the absence of ultrasound facilities: application in Indonesia
Source: BMC Pregnancy Childbirth. 2018 Nov 6;18:436. doi: 10.1186/s12884-018-2047-z (PMC6219176; doi:10.1186/s12884-018-2047-z)
Supplement: Supplementary file 7 — Table S7. Two independent sample t-tests between ABW, EFWr, and EFWp. Table S7 provides a two independent sample t-test to investigate if there is a significant difference between the observed or actual values of birth weight (ABW), recorded foetal weight estimation (EFWr), and estimated foetal weights based on the proposed model (EFWp). (PDF 157 kb) [file 12884_2018_2047_MOESM7_ESM.pdf]

Table S7 Two independent sample t-tests between ABW, EFW_r, and EFW_p

Sample size n = 38	Estimate for difference	DF	T-value	P-value
ABW – EFW_r	20.5	73	0.23	0.815
ABW – $EFW_{Model\ (1)}$	2.4	73	0.03	0.978
ABW – $EFW_{Model\ (2)}$	-0.2	73	-0.00	0.998
ABW – $EFW_{Model\ (3)}$	-1.6	73	-0.02	0.986
ABW – $EFW_{Model\ (4)}$	-0.3	73	-0.00	0.997

*The p-value < 0.05 indicates a significant difference
